# Autoimmune Conditions in 235 Hemochromatosis Probands with *HFE* C282Y Homozygosity and Their First-Degree Relatives

**DOI:** 10.1155/2015/453046

**Published:** 2015-10-04

**Authors:** James C. Barton, J. Clayborn Barton

**Affiliations:** ^1^Southern Iron Disorders Center, Birmingham, AL 35209, USA; ^2^Department of Medicine, University of Alabama at Birmingham, Birmingham, AL 35209, USA

## Abstract

We performed a retrospective study of autoimmune conditions (ACs) in 235 hemochromatosis probands at diagnosis by analyzing age, sex, ACs, history of first-degree family members with ACs (FH), diabetes, heavy ethanol consumption, elevated serum ALT/AST, nonalcoholic fatty liver disease, viral hepatitis, cirrhosis, iron removed to achieve iron depletion (QFe), and positivity for human leukocyte antigen (HLA) haplotypes A^*∗*^01, B^*∗*^08; A^*∗*^02, B^*∗*^44; A^*∗*^03, B^*∗*^07; A^*∗*^03, B^*∗*^14; and A^*∗*^29, B^*∗*^44. There were 138 men (58.7%). Median followup was 19.6 y. One or more of 19 ACs were diagnosed in each of 35 probands (14.9%). Prevalences of Hashimoto's thyroiditis, rheumatoid arthritis, and ankylosing spondylitis were 8.1% (95% CI: [5.1, 12.5]), 1.7% [0.6, 4.6], and 0.0085 [0.0015, 0.0337], respectively. Eighteen probands (7.7%) had a FH. Eight probands with ACs had 9 family members with ACs. In a logistic regression, ACs were less likely in men (odds ratio (OR) 0.3 [0.1, 0.6]) and more likely in probands with a FH (OR 4.1 [1.4, 11.8]). Overall ACs risk was not significantly associated with QFe or HLA haplotypes. Estimated survival of probands with and without ACs did not differ significantly. We conclude that ACs are common in hemochromatosis probands, especially women and probands with a FH.

## 1. Introduction

Hemochromatosis in whites of western European descent is an autosomal recessive disorder that increases the risk to develop iron overload. Severe iron overload can cause cirrhosis, primary liver cancer, diabetes mellitus, other endocrinopathies, and cardiomyopathy [1, 2]. By 1976, it was discovered that hemochromatosis in most whites is linked to human leukocyte antigen (HLA) A^*∗*^03 or HLA haplotypes A^*∗*^03, B^*∗*^07 or A^*∗*^03, B^*∗*^14 [3, 4]. For two decades thereafter, the combination of iron phenotyping and HLA-A and HLA-B typing was used to diagnose hemochromatosis in probands and first-degree family members [5].

In 1996, it was discovered that ~90% of whites with hemochromatosis were homozygous for a common mutation (exon 4, c.845G>A, p.C282Y; rs1800562) in a previously undescribed nonclassical class I major histocompatibility complex (MHC) gene on chromosome 6p [6]. The gene, now known as* HFE*, occurs in linkage disequilibrium with the HLA-A locus [3, 7, 8]. The most common hemochromatosis ancestral haplotypes in many northwestern European and derivative populations include* HFE* C282Y linked to HLA-A^*∗*^03 [7–10], HLA-A^*∗*^03, B^*∗*^07 [7–9], or HLA-A^*∗*^03, B^*∗*^14 [7–9, 11]. C282Y also occurs on other HLA haplotypes, including A^*∗*^01, B^*∗*^08 and A^*∗*^29, B^*∗*^44 [7, 8, 12, 13].

Autoimmune conditions (ACs) in persons with hemochromatosis have been documented predominantly in case reports, small case series, or studies of single ACs [14–19]. Our informal experience suggested that ACs are relatively common in adults with hemochromatosis. To study this further, we performed a retrospective analysis of ACs at diagnosis of hemochromatosis in 235 nonscreening probands with* HFE* C282Y homozygosity who had also undergone HLA-A and -B typing. Median followup was 19.6 y. We compared characteristics of probands with and without ACs, used univariable and multivariable methods to identify attributes significantly associated with ACs, and estimated survival of probands with and without ACs. We compiled a list of ACs previously reported in adults with hemochromatosis. We discuss our observations in the context of prevalence estimates of ACs in general populations and the association of ACs with serum ferritin (SF) and iron removed by phlebotomy to achieve iron depletion (QFe).

## 2. Methods

### 2.1. Selection of Hemochromatosis Probands

The performance of this work was approved by the Institutional Review Board of Brookwood Medical Center. Obtaining informed consent was not required because information reported herein was documented as part of routine medical care. We performed computerized and manual searches of medical records to identify patients who were evaluated for hemochromatosis in the interval 1976–2014 by the first author because they had elevated values of transferrin saturation or SF detected in medical care (not as part of family or population screening). Each patient selected for this study (a) was a white adult (>18 years of age) and the first in his/her respective family to be diagnosed to have hemochromatosis (proband); (b) had* HFE* C282Y/C282Y; (c) had undergone HLA-A and -B haplotyping; and (d) resided in central Alabama. Each proband was evaluated for iron overload and associated complications, as appropriate [20].

### 2.2. Autoimmune Conditions in Probands

We studied ACs diagnosed before hemochromatosis was diagnosed. Probands with ACs were characterized by referring physicians, our queries regarding ACs, medication reviews, interpretation of tissue specimens, and immunologic characteristics. In probands diagnosed to have Hashimoto's thyroiditis (HT), we concurred with diagnoses of HT after reviewing evidence of goiter or hypothyroidism, autoantibodies for thyroid peroxidase, thyroglobulin or thyroid-stimulating hormone receptors, features of thyroid glands typical of HT on pathology reports, use of thyroid hormone supplements, and exclusion or absence of other causes of hypothyroidism. Probands with rheumatoid arthritis had typical clinical manifestations, positive RA latex turbidity tests, and elevated levels of cyclic citrullinated peptide antibodies. We performed confirmatory tests for ACs in some probands.

### 2.3. Family History of Autoimmune Conditions

Reports of first-degree relatives (parents, full siblings, and children) with ACs (family history, FH) were elicited from each proband and documented at the time of initial evaluation for hemochromatosis. We interviewed first-degree relatives as part of family evaluations and reviewed their medical records or both.

### 2.4. Diabetes Mellitus in Probands

Probands with diabetes were diagnosed and characterized by referring physicians, our queries regarding diabetes, and medication reviews. Diabetes was defined and subclassified according to the criteria of the American Diabetes Association [21]. We excluded a woman with diabetes because she had undergone pancreatectomy for management of adenocarcinoma of the pancreas.

### 2.5. Definition of Heavy Ethanol Consumption

Heavy ethanol consumption was defined as the self-reported consumption of ≥60 g ethanol/d for ≥5 y [22].

### 2.6. Definitions of Liver Conditions

Elevated serum alanine aminotransferase (ALT) or aspartate aminotransferase (AST) was defined as a level >2 SD above mean (>40 IU/L). Nonalcoholic fatty liver disease (NAFLD) was defined as steatosis or steatohepatitis detected on liver biopsy specimens or by typical increase of hepatic echogenicity detected by ultrasonography, in the absence of self-reports of heavy ethanol consumption [22]. Chronic hepatitis B or C was defined as positivity for HB_s_Ag or hepatitis C antibody, respectively, in association with other clinical or liver biopsy abnormalities consistent with chronic viral hepatitis [22]. Liver biopsy was performed in probands with SF > 1000 *μ*g/L and in those suspected to have an undiagnosed non-iron liver disorder. Liver histology and intrahepatocytic iron were evaluated as previously described [22, 23]. Cirrhosis was defined by pathologists' interpretations of liver biopsy specimens [22].

### 2.7. Serum Ferritin and Iron Removed by Phlebotomy

SF values >300 *μ*g/L in men and >200 *μ*g/L in women were defined as elevated [20]. Iron depletion therapy, defined as the periodic removal of blood to eliminate storage iron, was complete when SF was ≤20 *μ*g/L [20]. QFe was estimated as 200 mg Fe per unit of blood (450–500 mL) [20].

### 2.8. Survival

All probands designated as alive were so confirmed on May 1, 2015. Dates of death were determined by review of medical records, by computerized searches for obituary notices, and by the Social Security Death Index (http://ssdi.rootsweb.ancestry.com/ and https://www.dobsearch.com/death-records/search-ssn.php). Survival after diagnosis was computed using date of diagnosis of hemochromatosis and either May 1, 2015, or date of death, as appropriate. Overall survival was computed using date of birth and either May 1, 2015, or date of death, as appropriate.

### 2.9. Laboratory Methods

Levels of SF, ALT, AST, HB_s_Ag, HB_s_Ab, HB_c_Ab, and hepatitis C antibody were measured using automated clinical methods.* HFE* mutation analysis was performed as previously described [24]. HLA-A and -B alleles were detected using low-resolution DNA-based typing in probands and family members; haplotypes were ascertained using HLA analyses of appropriate first-degree family members as previously described [7].

### 2.10. Statistics

SF and QFe values were converted to natural logarithms (ln) to normalize them for analyses; antilns were computed to display mean values (95% confidence intervals (CI)). The Pearson correlation coefficient of lnSF with lnQFe was 0.4509 (two-tailed test; *p* < 0.0001). We used QFe as the independent variable representing iron overload severity. Phlebotomy data were incomplete in 20 probands (14 men and 6 women; 8.5%) and thus their QFe data were not used for analyses.

The final analytic data set consisted of observations on 235 probands. Logistic regressions were performed to identify independent variables significantly associated with ACs and HT. We did not use logistic technique to assess predictors of rheumatoid arthritis, ankylosing spondylitis, or other ACs because logistic models suffer from small sample bias [25]. Independent variables appropriate for most regression analyses were age, sex, FH, diabetes mellitus, heavy ethanol consumption, elevated ALT/AST, NAFLD, viral hepatitis, cirrhosis, QFe, and positivity for HLA haplotypes A^*∗*^01, B^*∗*^08; A^*∗*^02, B^*∗*^44; A^*∗*^03, B^*∗*^07; A^*∗*^03, B^*∗*^14; and A^*∗*^29, B^*∗*^44. We used backward stepwise multiple regression to identify predictors of SF. We used Kaplan-Meier technique (log-rank test) to estimate survival and Cox proportional hazards models to identify significant contributors to death.

General descriptive data are presented as enumerations, percentages, mean ±1 standard deviation (SD), or mean [95% CI]. Univariable comparisons between groups were evaluated using Student's *t*-test, Pearson's *X* ^2^ test, and Fisher's exact test, as appropriate. We expressed some results as odds ratios (OR [95% CI]). We computed the 95% CI of proportions with continuity corrections. Values of *p* < 0.05 were defined as significant. Bonferroni corrections were applied to control type I error rate at 0.05 for multiple comparisons of continuous and dichotomous data, as appropriate. Analyses were performed with a computer spreadsheet (Excel 2000, Microsoft Corp., Redmond, WA) and a statistical program (GB-Stat, v. 10.0, 2000, Dynamic Microsystems, Inc., Silver Spring, MD).

### 2.11. Literature Search

We performed a computerized search of the National Library of Medicine database to identify previous reports of ACs in persons with hemochromatosis. We used two terms per search: the first was the name of an AC and the second was hemochromatosis. Reports were reviewed to verify the diagnosis of hemochromatosis defined as an adult-onset condition typical of* HFE* hemochromatosis in whites of western European descent characterized by iron phenotyping, HLA typing or haplotyping, or* HFE* C282Y homozygosity.

## 3. Results

### 3.1. General Characteristics of 235 Probands

The mean age at diagnosis of hemochromatosis was 49 ± 13 y. There were 138 men (58.7%). One or more ACs were diagnosed in each of 35 probands (14.9%; [10.7, 20.2]). The proportion of men was lower among 35 probands with ACs than among 200 probands without ACs (Table 1). Eighteen probands (7.7%; [4.7, 12.0]) had a FH of ACs. The proportion of probands with a FH was greater among those with ACs (Table 1). Prevalences of other characteristics did not differ significantly between probands with and without ACs (Table 1). The previous diagnosis of primary biliary cirrhosis was confirmed in one of 102 probands who underwent liver biopsy at the time of hemochromatosis diagnosis. No proband was diagnosed to have anterior pituitary or myocardial siderosis.

Mean SF at diagnosis of hemochromatosis was 761 *μ*g/L [677, 858]. SF > 300 *μ*g/L was observed in 131 men (94.9%). Of these, 124 (89.9%) achieved iron depletion. SF > 200 *μ*g/L was observed in 86 women (88.7%). Of these, 91 (93.8%) achieved iron depletion. Phlebotomy data were incomplete in 20 probands (Table 1). Mean QFe in 215 probands was 2.0 g [1.7, 2.3]. Mean QFe in probands with and without ACs did not differ significantly (Table 1).

### 3.2. Autoimmune Conditions in 35 Probands

Nineteen different ACs were diagnosed in 35 probands. HT was diagnosed in 19 of 35 probands with ACs (54.3%) (Table 2). The female: male ratio among probands with HT was 2.8. The prevalence of HT was greater in women than men (14.4% versus 3.6%, resp.; *p* = 0.0031; OR 4.6 [1.6, 13.1]). Positivity for HLA-A^*∗*^03 or HLA haplotypes did not differ significantly between 19 probands with HT and 216 probands without HT (data not shown). Among 216 probands without HT, 16 (7.4%; [4.4, 12.0]) had one or more ACs. No proband was diagnosed to have an autoimmune polyendocrine syndrome or diabetes mellitus type 1.

### 3.3. Ankylosing Spondylitis and HLA-**B** ^**∗**^27 Positivity

Heterozygosity for B^*∗*^27 was detected in 16 probands (6.8%). Homozygosity for B^*∗*^27 was not observed. Neither of two probands with ankylosing spondylitis was positive for B^*∗*^27. Only one proband with an AC, a man with HT, was positive for B^*∗*^27. Positivity for B^*∗*^27 in the present 235 probands and in 1318 central Alabama white control subjects [7] did not differ significantly (0.0681 versus 0.0895, resp.; *p* = 0.2808). Eight probands had either two B^*∗*^07 alleles, two B^*∗*^14 alleles, or one of each, as part of ancestral hemochromatosis haplotypes. Accordingly, only 227 probands would have been “at risk” for inheritance of B^*∗*^27. Even with this correction, the difference in positivity for B^*∗*^27 in 227 probands and in 1318 control subjects was not significant (0.0705 versus 0.0895, resp.; *p* = 0.3464).

### 3.4. Autoimmune Conditions in First-Degree Family Members

Eighteen probands had 19 family members with ACs, among which HT was the most prevalent (Table 3). The proportion of female relatives with ACs (16/19; 84.2%) was greater than the proportion of male relatives with ACs (3/19; 15.8%) (*p* < 0.0001).

Eight probands with ACs had 9 family members who also had ACs. There was concordance for specific ACs in at least two generations in 7 of the 8 kinships: HT (four kinships) and Crohn's disease, Graves' disease, and sarcoidosis (one kinship each). In one kinship, both a female proband and her mother, also a C282Y homozygote, had HT.

### 3.5. Predictors of Autoimmune Conditions

We performed an initial regression on ACs using 16 appropriate independent variables. In a refined model that included only two variables, there was a negative association of male sex with ACs (*p* = 0.0010; OR 0.3 [0.1, 0.6]). There was a positive association of FH with ACs (*p* = 0.0093; OR 4.1 [1.4, 11.8]). This 2-factor model explained 11.0% of total deviance contributing to ACs (*X* ^2^ = 21.69; *p* < 0.0001).

### 3.6. Predictors of Hashimoto's Thyroiditis

We performed an initial regression on HT using 16 appropriate independent variables. The variables age, male sex, FH, and QFe were used in a final model. There was a negative association with male sex (*p* = 0.0216; OR 0.3 [0.1, 0.8]). There was a positive association of HT with FH (*p* = 0.0323; OR 3.8 [1.1, 13.0]). This 4-factor model explained 13.0% of total deviance contributing to HT (*X* ^2^ = 17.16; *p* = 0.0018).

### 3.7. Predictors of Autoimmune Conditions other than Hashimoto's Thyroiditis

We performed a logistic regression on ACs after removing observations on 19 probands with HT from the analytic dataset. An initial regression included 16 appropriate independent variables. Age, male sex, FH, and QFe were appropriate for a final model. The only significant (negative) predictor was male sex (*p* = 0.0261; OR 0.3 [0.1, 0.9]). This 4-factor model explained 9.7% of total deviance contributing to ACs other than HT (*X* ^2^ = 11.12; *p* = 0.0252).

### 3.8. Predictors of Serum Ferritin

We performed a backwards stepwise regression on SF using 16 independent variables. There were significant positive associations with male sex (*p* < 0.0001); diabetes (*p* = 0.0403); elevated AST (*p* = 0.0375); and cirrhosis (*p* < 0.0001). There was a significant negative association with HLA-A^*∗*^01, B^*∗*^08 (*p* = 0.0047). This 5-factor model explained 23.6% of total deviance contributing to SF (ANOVA *p* = 0.0252).

### 3.9. Survival

Median survival after diagnosis of hemochromatosis was 19.6 y (mean 18.5 ± 7.7 y). Median overall survival was 68.0 y (mean 66.8 ± 14.8 y). There were 40 deaths (27 men, 13 women) (17.0%). No death was attributed to direct or indirect consequences of ACs.

**Figure 1:**
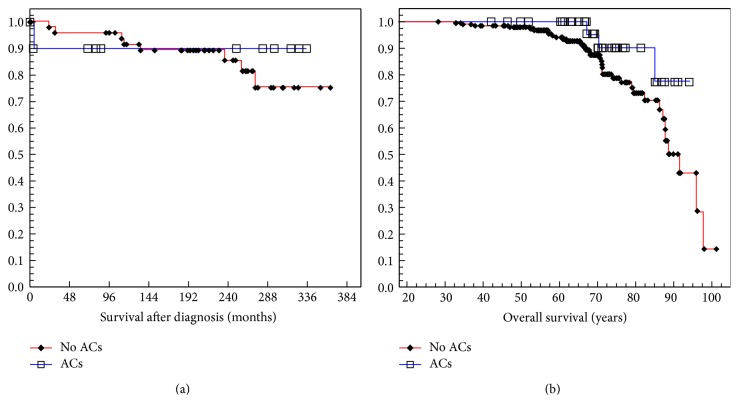
(a) Kaplan-Meier estimates of survival after hemochromatosis diagnosis in probands with and without autoimmune conditions (ACs) (*p* = 0.6380; log-rank test). (b) Kaplan-Meier estimates of overall survival of hemochromatosis probands with and without ACs (*p* = 0.1127; log-rank test).

Kaplan-Meier estimates of survival after hemochromatosis diagnosis of probands with and without ACs were similar (*p* = 0.6380) (Figure 1). Cox proportional hazards analysis revealed two positive associations with death: age (*p* = 0.0107) and QFe (*p* = 0.0032). Overall survival of probands with and without ACs was also similar (*p* = 0.1127) (Figure 1). Cox proportional hazards analysis revealed two positive associations with death: heavy ethanol consumption (*p* = 0.0228) and cirrhosis (*p* = 0.0096). Comparison of survival after hemochromatosis diagnosis or overall survival of men with and without ACs and of women with and without ACs revealed no significant differences (data not shown).

### 3.10. Autoimmune Conditions Previously Reported in Persons with Hemochromatosis

These ACs are displayed in Table 4.

## 4. Discussion

The present results demonstrate that 14.9% of 235 hemochromatosis probands with* HFE* C282Y homozygosity had one or more of 19 different ACs at diagnosis of hemochromatosis. ACs were more likely to occur in women and in probands with first-degree relatives who had ACs. HT was diagnosed in 54% of probands with ACs. Among 216 probands without HT, 7.4% had ACs that were positively associated with women. Overall risk of ACs was not significantly associated with QFe. ACs were not significantly associated with positivity for HLA-A^*∗*^03 or HLA-A and -B haplotypes, including the A^*∗*^01, B^*∗*^08 haplotype associated elsewhere with ACs [46]. Estimated survival of probands with and without ACs did not differ significantly.

Population prevalence rates for ACs vary for many reasons [47], including underrepresentation of certain ACs in study cohorts [47, 48], high prevalence of undiagnosed ACs [49], lack of prevalence data for some ACs [47], and differences across race/ethnicity groups or geographic regions [48]. The combined prevalence of 24 ACs in persons unselected for hemochromatosis diagnoses was 3.2% [50]. The combined prevalence of 31 ACs in hospitalized Danes was more than 5% [48]. In 2009, Cooper and colleagues, using “adjustments” to these prevalence assessments [48, 49], estimated that the overall population prevalence of ACs is 9.4% [47]. These observations suggest that the combined prevalence of ACs in the present probands of 14.9% (10.7, 20.2) is similar to that in populations not selected for hemochromatosis diagnoses.

The prevalence of HT in the present probands was 8.1% (5.1, 12.5). In women aged 20–49 y in a large population hemochromatosis screening program in Norway, 12.5% of* HFE* C282Y homozygotes and 3.0% of control participants reported having hypothyroidism [51]. In the Hemochromatosis and Iron Overload Screening Study of adult primary care participants in North America, 8.5% of 176* HFE* C282Y homozygotes and 10.9% of 312* HFE*  wt/wt controls reported taking thyroid supplements (*p* = 0.4019) [52]. Prevalence estimates for untreated hypothyroidism did not differ significantly between C282Y homozygotes (1.7%) and controls (1.3%) or between male and female C282Y homozygotes and corresponding controls [52]. The prevalence of HT confirmed by cytology in 811 consecutive patients unselected for hemochromatosis diagnoses who underwent fine-needle aspiration of thyroid nodules was 13.4%, of whom 5.7% (4.1, 7.4) were clinically hypothyroid [53]. Although some differences in prevalence estimates of HT or presumed HT are due to dissimilarities in study design and case ascertainment, we infer that prevalences of HT in hemochromatosis probands diagnosed in medical care and in* HFE* C282Y homozygotes identified in screening are similar to those in other populations [53].

HT was significantly associated with the present female probands in univariable and multivariable analyses. The female: male ratio of HT was 2.8. In other studies, female: male ratios of autoimmune thyroiditis were 5.1–9.1 [54]. In a meta-analysis, skewed X-inactivation was significantly greater in women with HT than in control subjects. This could explain in part the preponderance of women in HT cohorts [55]. On the other hand, English gerontologist Joseph Sheldon did not describe thyromegaly, lymphocytic infiltrates, or other attributes of autoimmune thyroiditis in his 1935 monograph of 311 hemochromatosis cases [56]. It is plausible that gross and microscopic manifestations typical of autoimmune thyroiditis were obscured by thyroid fibrosis attributed to iron deposits or were not detected because women represented only 5% of the cases [56].

The prevalence of rheumatoid arthritis in the present probands (1.7% [0.6, 4.6]) is similar to that in the U.S. general adult population (0.5–1.0%) [57, 58]. Some manifestations of hand arthropathy due to hemochromatosis and rheumatoid arthritis are similar [59]. To distinguish these conditions, it is prudent to perform an immunologic evaluation for rheumatoid arthritis in patients with hemochromatosis and hand arthropathy [40, 41, 59].

The prevalence of ankylosing spondylitis in the present probands (15–337/10,000) is similar to that of 32/10,000 in North American subjects unselected for hemochromatosis or* HFE* C282Y homozygosity [60]. HLA-B^*∗*^27 positivity rates in the present probands, in white control subjects from central Alabama [7], and in Caucasian control subjects in the US [61] are similar (7–9%). Neither of the two present probands diagnosed to have ankylosing spondylitis was positive for B^*∗*^27, whereas 80–95% of persons with ankylosing spondylitis worldwide are B^*∗*^27-positive [62]. We attribute this difference to the small sample size of probands with ankylosing spondylitis in the present study. There are radiographic similarities and dissimilarities in spine manifestations of ankylosing spondylitis and hemochromatosis [63].

QFe was not significantly associated with ACs in the present study. In contrast, the prevalence of autoimmune thyroid disorders in 34 men homozygous for hemochromatosis alleles (8.8%) was higher than in the general male population [16]. Although it has been postulated that iron deposits in the thyroid glands of persons with hemochromatosis cause HT or Graves' disease [16, 31, 64], this postulate remains unproven. Iron deposits were detected in the thyroid glands of a majority of persons with hemochromatosis studied at autopsy [56, 64, 65]. Among 391 persons with hemochromatosis diagnosed in medical care, only 4.1% had primary hypothyroidism and only 0.3% had hyperthyroidism [16, 66–69]. Thus, it is unlikely that iron deposits in the thyroid gland contribute to the pathogenesis of hypothyroidism or hyperthyroidism in most persons with hemochromatosis. There are few well-documented hemochromatosis patients who had hypothyroidism due to impaired thyrotroph function. Most of them also had hypogonadotropic hypogonadism [70–74]. Iron deposits were visualized in a minority of thyrotrophs in some persons with severe iron overload due to hemochromatosis. The deposits were much less prominent than those in gonadotrophs [64, 75]. In rare cases, secondary hypothyroidism resolved after iron depletion [72, 74].

There was intraperson co-occurrence of two or more ACs in six probands, five of whom had HT. Persons with HT unselected for hemochromatosis diagnoses also have increased risk for co-occurrence of other ACs, including Addison's disease [76, 77]; autoimmune hepatitis [78]; celiac disease [76, 79]; diabetes mellitus type 1 [80–82]; mixed connective tissue disorder [83]; multiple sclerosis [33]; pernicious anemia [76, 77]; polymyositis/dermatomyositis [83]; rheumatoid arthritis [33, 83]; Sjögren's syndrome [83]; systemic lupus erythematosus [76, 83]; systemic sclerosis [83]; and vitiligo [76]. One of the present probands, a woman, had both ulcerative colitis and biliary cirrhosis. Co-occurrence of these conditions in the same individual is uncommon but well documented [84–86].

There was co-occurrence of specific ACs in two (or three) generations in seven of the present kinships, including four kinships with HT. Familial clusters of specific ACs occur, depending upon the proband's specific AC [47, 87]. There is strong familial clustering of autoimmune thyroid disease in kinships unselected for hemochromatosis diagnoses [88, 89]. It is also more feasible to detect statistically significant associations with autoimmune thyroid conditions than associations with ACs that are less common [47].

SF levels were elevated in 92% of the present probands and in some patients with ACs who were not selected for hemochromatosis diagnoses [90]. ACs were not significant predictors of SF in the present study. SF is a mixture of iron-rich ferritin and apoferritin [91, 92]. The iron composition of SF is increased in hemochromatosis and other iron overload disorders, consistent with the function of ferritin as an iron storage protein [91, 93]. It cannot be determined whether increased amounts of apoferritin were released into the blood of some of the present probands as acute phase reactants due to inflammation or interleukin-1 [92, 94].

Survival estimates in probands with and without ACs did not differ significantly. Although ACs are among the leading causes of death in young and middle-age women in the US [95], death of none of the 40 present probands was attributed to consequences of ACs. Decreased survival after diagnosis of hemochromatosis was significantly associated with age at diagnosis and QFe. Decreased overall survival was significantly associated with heavy ethanol consumption and cirrhosis. These outcomes are consistent with previous reports of survival of patients with hemochromatosis unselected for diagnoses of ACs [11, 22, 96].

ACs in two or more previous reports (HT, Graves' disease, rheumatoid arthritis, pernicious anemia, and sarcoidosis) mirror the relative prevalences of the corresponding ACs in the present probands. We may have missed other reports of ACs in persons with hemochromatosis because the search terms we used did not correspond to the key words for indexing the reports. There are multiple reports of the concurrence of hemochromatosis and celiac disease [17, 19, 26–29]. Although hemochromatosis was associated with an increased risk of celiac disease in one study [19], none of the present probands was diagnosed to have celiac disease.

There are uncertainties in the present results. Our enumerations and prevalence estimates of ACs are conservative because some probands may have failed to report or their medical records did not document that they had ACs or FH of ACs. Some persons with HT are euthyroid or have subclinical hypothyroidism [54] and thus their thyroiditis may have been unrecognized at the time of hemochromatosis diagnosis. Followup of each proband for the possible development of ACs after hemochromatosis diagnosis was beyond the scope of the present study, although we observed informally that two probands developed HT several years after they achieved iron depletion. Many ACs have been described in insufficient numbers of persons with hemochromatosis to permit meaningful prevalence estimates. The reliability of FH reports in previous studies of ACs [88, 89], especially when medical records were available [97], was excellent. Complete QFe data were not available in 20 of the present probands. Based on the results of univariable comparisons and regression analyses of QFe observations in the other 215 probands, it is unlikely that the present results would have differed significantly had QFe data been available for all probands. The present observations cannot exclude the possibility that MHC-linked genes, including some loci linked to HLA-A and -B [46, 98], contributed to the pathogenesis of ACs in the present probands. HLA characteristics of* HFE* C282Y homozygotes that reside in other geographic areas may differ from those of the present probands. It is plausible but unproven that diagnosis and management of ACs increase the likelihood that a* HFE* C282Y homozygote would be diagnosed subsequently to have hemochromatosis.

## 5. Conclusions

We conclude that ACs are common in hemochromatosis probands, especially women and probands with a FH. Overall risk of ACs is not significantly associated with QFe or HLA haplotypes.

## Figures and Tables

**Table 1:** Comparisons of hemochromatosis probands with and without autoimmune conditions^1^.

Characteristic	Autoimmune condition (*n* = 35)	No autoimmune condition (*n* = 200)	Value of *p* ^2^
Mean age, y	53 ± 13	48 ± 13	0.0747
Men, %	28.6 (10)	64.0 (128)	<0.0001
Family history, % (*n*)^3^	22.9 (8)	5.0 (10)	0.0002
Diabetes mellitus, % (*n*)	14.3 (5)	14.0 (28)	0.9642
Heavy ethanol, % (*n*)	5.7 (2)	14.0 (28)	0.1754
Elevated ALT, % (*n*)^4^	20.0 (7)	25.0 (50)	0.5243
Elevated AST, % (*n*)^4^	25.7 (9)	28.0 (56)	0.7803
NAFLD, % (*n*)	17.1 (6)	19.0 (38)	0.7950
Viral hepatitis, % (*n*)	2.9 (1)	5.0 (10)	0.4922
Cirrhosis, % (*n*)	14.3 (5)	10.0 (20)	0.4481
Mean SF, *µ*g/L	551 [72, 4202]	807 [133, 4879]	0.0441
Mean FeQ, g^5^	1.7 [1.1, 2.5]	2.0 [1.7, 2.4]	0.3962
HLA-A^*∗*^03 positivity, % (*n*)	77.1 (27)	71.5 (143)	0.4911
HLA-A^*∗*^01, B^*∗*^08 positivity, % (*n*)	14.3 (5)	11.0 (22)	0.5739
HLA-A^*∗*^02, B^*∗*^44 positivity, % (*n*)	11.4 (4)	9.0 (18)	0.4911
HLA-A^*∗*^03, B^*∗*^07 positivity, % (*n*)	48.6 (17)	41.0 (82)	0.4026
HLA-A^*∗*^03, B^*∗*^14 positivity, % (*n*)	22.9 (8)	15.5 (31)	0.2805
HLA-A^*∗*^29, B^*∗*^44 positivity, % (*n*)	0	5.5 (11)	0.1626

^1^ALT, alanine aminotransferase; AST, aspartate aminotransferase; NAFLD, nonalcoholic fatty liver disease; SF, serum ferritin; FeQ, iron removed by phlebotomy to achieve iron depletion; HLA, human leukocyte antigen. Mean results are displayed as mean ± SD or mean [95% CI].

^2^These are nominal values of *p*. Bonferroni correction for 18 comparisons yielded a revised *p* for significance of <0.0028.

^3^History of autoimmune condition(s) in one or more first-degree family members.

^4^Elevated values were defined as >40 IU/L.

^5^These observations represent 215 probands because phlebotomy data were incomplete in 20 probands.

**Table 2:** Autoimmune conditions in 35 hemochromatosis probands^1^.

Condition	Percentage (*n*)
Hashimoto's thyroiditis^2^	54.3 (19)
Rheumatoid arthritis^3^	11.4 (4)
Ankylosing spondylitis^4^	5.7 (2)
Mixed connective tissue disorder	5.7 (2)
Myasthenia gravis	5.7 (2)
Pernicious anemia	5.7 (2)
Sarcoidosis	5.7 (2)
Ulcerative colitis	5.7 (2)
Autoimmune hemolytic anemia^5^	2.9 (1)
Crohn's disease	2.9 (1)
Elevated antinuclear antibody^6^	2.9 (1)
Graves' disease	2.9 (1)
Polymyalgia rheumatica	2.9 (1)
Primary biliary cirrhosis	2.9 (1)
Psoriasis	2.9 (1)
Raynaud's phenomenon	2.9 (1)
Scleroderma	2.9 (1)
Sjögren's syndrome	2.9 (1)
Systemic lupus erythematosus	2.9 (1)

^1^Six probands had two or more autoimmune conditions. Two men had both Hashimoto's thyroiditis and pernicious anemia. One woman had both Hashimoto's thyroiditis and Crohn's disease. One woman had Hashimoto's thyroiditis, polymyalgia rheumatica, and systemic lupus erythematosus. One woman had Hashimoto's thyroiditis, Raynaud's phenomenon, sarcoidosis, scleroderma, and Sjögren's syndrome. One woman had both ulcerative colitis and biliary cirrhosis.

^2^Of 235 probands, 14 women and 5 men had Hashimoto's thyroiditis (8.1% [5.1, 12.5]).

^3^Of 235 probands, 2 men and 2 women had rheumatoid arthritis (1.7% [0.6, 4.6]).

^4^Of 235 probands, 2 men had ankylosing spondylitis (0.0085 [0.0015, 0.0337]).

^5^Mediated by IgG and complement.

^6^This proband had anti-nuclear antibody >1 : 320 without other manifestation of autoimmunity. Elevated anti-nuclear antibodies (>1 : 80) in other probands were interpreted as part of broader autoimmune condition diagnoses named separately.

**Table 3:** Autoimmune conditions in 18 first-degree relatives of 235 hemochromatosis probands^1^.

Conditions in relatives	35 probands with autoimmune conditions	200 probands without autoimmune conditions	Value of *p* ^2^
Hashimoto's thyroiditis	11.4 (4)	0.5 (1)	0.0019
Crohn's disease	5.7 (2)	0	0.0216
Graves' disease	2.9 (1)	0.5 (1)	0.2762
Rheumatoid arthritis	2.9 (1)	2.0 (4)	0.5569
Sarcoidosis	2.9 (1)	0	0.1489
Vitiligo	2.9 (1)	0	0.1489
Addison's disease	0	0.5 (1)	0.8511
Multiple sclerosis	0	0.5 (1)	0.8511
Pernicious anemia	0	0.5 (1)	0.8511
Scleroderma	0	0.5 (1)	0.8511

^1^Observations are displayed as % (*n*) of the proband subgroups who had first-degree relatives with the indicated conditions. Each of two relatives had two autoimmune conditions.

^2^These are nominal values of *p*. Bonferroni correction for 10 comparisons yielded a revised *p* for significance of <0.0050.

**Table 4:** Autoimmune conditions previously reported in persons with hemochromatosis^1^.

Condition	Reference
Autoimmune hemolytic anemia	[14]
Celiac disease	[17, 19, 26–29]
Diabetes mellitus type 1^2^	[30]
Hashimoto's thyroiditis	[16, 31, 32]
Hyperthyroidism or Graves' disease	[16, 33–35]
Immune thrombocytopenia	[34]
Myasthenia gravis^3^	[36]
Pernicious anemia	[37, 38]
Psoriasis	[39]
Rheumatoid arthritis	[40, 41]
Sarcoidosis^4^	[18, 42]
Schnitzler's syndrome	[43]
Sclerosing cholangitis^5^	[44]
Ulcerative colitis^5^	[44]
Vitiligo	[15]

^1^Hemochromatosis was defined as an adult-onset condition typical of *HFE* hemochromatosis in European white adults characterized by iron phenotyping, HLA typing or haplotyping, or *HFE* C282Y homozygosity. One *HFE *C282Y homozygote had collagenous sprue, a rare condition of uncertain etiology [45].

^2^Autoimmunity was not mentioned in descriptions of study subjects.

^3^This man had pure red cell aplasia of undefined cause.

^4^The case of a woman with sarcoidosis included herein was reported previously [18].

^5^Ulcerative colitis and sclerosing cholangitis occurred in the same man.

## Abbreviations

ACs: Autoimmune conditions
ALT: Serum alanine aminotransferase
AST: Serum aspartate aminotransferase
CI: Confidence interval
FH: History of autoimmune conditions in first-degree family members
HLA: Human leukocyte antigen
HT: Hashimoto's thyroiditis
MHC: Major histocompatibility complex
OR: Odds ratio
QFe: Quantity of iron removed by phlebotomy to achieve iron depletion
SD: Standard deviation
SF: Serum ferritin.
